# Hidden Asymmetries: Leg Length Discrepancy and Breast Asymmetry in Adolescent Scoliosis and Postural Disorders—A Cross-Sectional Study

**DOI:** 10.3390/jcm14113793

**Published:** 2025-05-28

**Authors:** Nicola Manocchio, Roberta Marini, Concetta Ljoka, Laura Giordani, Isabella Iovene, Giulia Vita, Calogero Foti

**Affiliations:** 1 Physical and Rehabilitation Medicine, Tor Vergata University, 00133 Rome, Italy; roberta.marini88@gmail.com (R.M.); concetta.ljoka@ptvonline.it (C.L.); laura.giordani@uniroma2.it (L.G.); isabellaiovene89@gmail.com (I.I.); giulia.vita@students.uniroma2.eu (G.V.); foti@med.uniroma2.it (C.F.); 2 PhD Course in Tissue Engineering and Remodeling Biotechnologies for Body Function, Tor Vergata University, 00133 Rome, Italy

**Keywords:** scoliosis, leg length inequality, breast asymmetry, rehabilitation, quality of life, posture

## Abstract

**Background/Objectives:** Morphological spinal alterations in adolescents, including idiopathic scoliosis (IS) and postural scoliotic attitudes (paramorphisms), may be associated with leg length discrepancy (LLD) and breast asymmetry (BA). This study aimed to assess the prevalence and characteristics of LLD and BA in adolescents with spinal paramorphisms and dysmorphisms (IS), and to explore associations between these asymmetries and spinal curve features. **Methods**: A cross-sectional observational study was conducted. Adolescents aged 10–18 years were included. LLD was measured clinically via direct tape measurement and, when necessary, ultrasound. BA was assessed via visual inspection. Spinal deformities were characterized via clinical and radiological examination. **Results**: Among the 44 participants, 26 (60%) had IS and 18 (40%) had postural scoliotic attitudes. LLD was present in 79.5% (mean 0.7 ± 0.6 cm; all mild). BA was observed in 14% of the sample. LLD was more frequent in IS (87%) than in postural scoliotic attitudes (72%). In lumbar postural curves, the shorter limb was consistently ipsilateral to the curve convexity. In IS, no consistent association was found between LLD and curve characteristics. BA was slightly more prevalent in IS (19%) than postural scoliotic attitudes (17%), with no consistent pattern relative to curve convexity. **Conclusions**: Mild LLD is common in adolescents with spinal asymmetries and reflects general population norms. While LLD may influence compensatory postural curves, it does not appear to affect IS curve patterns or severity. BA is more frequent in IS, but shows variable association with curve features. Considering LLD and BA prevalence in adolescents with spinal asymmetries routine assessment is warranted, though their impact on IS progression is limited.

## 1. Introduction

Morphological alterations of the spine can be clinically categorized into paramorphisms and dysmorphisms [1]. Paramorphisms, characterized by their transitory and reversible nature, refer to morphological deviations of the spine caused by improper postural habits, not supported by structural skeletal alterations. Paramorphisms result from maintaining incorrect positions over time and can often be corrected through postural adjustments or physiotherapy. Examples include slight lateral curvatures of the spine, such as scoliotic posture, which do not involve permanent structural changes [2]. Dysmorphisms, on the other hand, involve structural alterations of the vertebral bodies that lead to permanent morphological changes in the spine. These conditions are often congenital or acquired and are not correctable either spontaneously or actively. A typical example of a dysmorphism is scoliosis, where vertebral rotation and deformation occur. Dysmorphisms require more complex treatment approaches, including motor re-education, bracing, or surgery [3]. Scoliosis is a structural abnormality of the spine involving all three spatial planes. In the frontal plane, it presents as a lateral flexion or curvature of the spine. In the sagittal plane, it involves alterations of the spine’s physiological curves, such as hypokyphosis or hyperlordosis. In the transverse plane, scoliosis is characterized by vertebral rotation, which contributes to its three-dimensional nature and often results in rib cage abnormalities [4]. In the scientific literature, scoliosis is universally defined as a spinal curvature exceeding 10° Cobb in the coronal plane [5]. Idiopathic scoliosis (IS) is the most common form of scoliosis. IS prevalence ranges between 0.47% and 5.2%, with a higher incidence in females. The female-to-male ratio is 1.5:1 for mild cases, but this disparity increases significantly to 10:1 in severe cases, defined as those with a Cobb angle greater than 30° [6]. Scoliosis typically develops and progresses without symptoms, posing the risk that patients may seek specialist care only when the scoliotic curve deformity becomes visibly apparent [7,8]. Clinical assessment involves evaluating shoulder alignment and scapular symmetry [9]. For the lower limbs, the alignment of the iliac crests or anterior superior iliac spines is examined to identify potential leg length discrepancies (LLDs). Assessing limb length discrepancy is crucial, as it can negatively affect pelvic positioning and lead to frontal plane imbalance [10,11]. The Adams Forward Bend Test is applied to evaluate rib cage abnormalities. A positive test result is indicated when, during forward flexion, one side of the back appears higher than the contralateral side, suggesting vertebral rotation and confirming the presence of scoliosis [12]. The final confirmation is through radiological examination and Cobb angle measurement [13].

The term LLD refers to a difference in length between the two limbs, and is a condition frequently observed in both pediatric and adult populations. Based on the extent of the difference in the lower limb length, three categories of limb length discrepancy have been identified: mild (< 3 cm), moderate (≥ 3 cm and ≤ 6 cm), and severe (> 6 cm) [14]. Typically, the degree of discrepancy is mild and remains asymptomatic in affected individuals [15]. Structural LLDs are associated with the shortening of the bony structures that make up the lower limb, which may result from fractures, congenital or acquired bone deformities, or abnormalities in the physiological growth process of the limb or hip joint [16,17,18]. On the other hand, functional limb length discrepancies are associated with alterations in the mechanics of the lower limbs, such as joint limitations, static or dynamic misalignment of the mechanical axis, muscle weakness, or muscle shortening. Pelvic abnormalities can also contribute to functional discrepancies, as seen in cases of pelvic rotations or tilts caused by ligament shortening or muscular fascia contractures [19]. The clinical assessment of LLD employs two methods: direct and indirect. The direct method is performed with the patient in a supine position, using a tape measure to determine the distance between fixed bony landmarks. The most commonly used measurement is the distance between the anterior superior iliac spine and the medial malleolus [20]. The indirect method is performed with the patient in a standing position and involves assessing pelvic alignment using the iliac crests as reference points. If a difference in the height of the iliac crests is observed, one or more lifting blocks of varying sizes are placed under the foot of the shorter leg until the pelvis and iliac crests are properly aligned [10].

LLD can lead to postural alterations and gait asymmetry, related to biomechanical compensations. These include knee and hip flexion contractures on the side of the longer limb, plantar flexion of the ankle, a reduction in the hip adduction angle, and an increase in the external rotation of the trunk and pelvis on the side of the shorter limb [21,22,23]. In the longer limb, foot pronation often occurs, while in the shorter limb, supination and/or plantar flexion of the foot may be observed. The knee and hip also contribute to compensation, typically through flexion in the longer limb and extension in the shorter limb. At the pelvic level, if the compensatory mechanisms of the lower limbs are insufficient, the anterior and posterior iliac spines tilt downward on the side of the shorter limb. This can result in a sacral base tilt, potentially leading to functional scoliosis [24].

Recently, IS has been associated with breast asymmetry (BA) [25]. BA is defined as a difference in the shape, volume, and/or position of the breast or the nipple-areola complex [26]. The majority of women exhibit mild physiological BA, with the left breast typically being slightly larger than the right [27]. Several studies have shown that BA is significantly more common in adolescents with thoracic IS, with the breast located on the convex side of the scoliotic curve showing reduced volume, a more cranial breast base position, a shorter sternal–breast distance, and a smaller areola compared to the contralateral side. [25,28,29,30]. The underlying causes of this asymmetry remain unclear to date. Some authors have focused on the anterior rib hump secondary to the thoracic curve, but no statistically significant correlation has been identified between anterior rib prominence and breast asymmetry [31]. Other authors hypothesize that asymmetric blood flow from the internal mammary artery, which supplies the anterior thoracic wall, may influence breast development. They propose that the use of corrective trunk orthoses could induce intermittent compression of this vessel, potentially contributing to altered blood flow patterns [32]. Regarding the relationship between BA, curve type, and severity (i.e., Cobb angle), no conclusive consensus exists in the literature. Tsai et al. [33] suggest that the volume difference between breasts is significantly correlated with scoliosis severity, while Shi et al. assert that BA is independent of the degree of vertebral rotation [30].

The aim of this work was to investigate the prevalence and characteristics of LLD and BA in adolescents presenting with spinal paramorphisms (postural scoliotic attitudes) and dysmorphisms (IS), and to explore potential associations between LLD, BA, and the type, location, and laterality of spinal curvature.

## 2. Materials and Methods

### 2.1. Study Design and Participants

This cross-sectional observational study was conducted on consecutive patients starting in February 2020 through December 2024 at the Physical Medicine and Rehabilitation outpatients Unit of the Tor Vergata University Hospital, Rome, Italy. Reporting has been carried out following the STROBE checklist [34].

The protocol was conducted, recorded, and reported following the Good Clinical Practice guidelines and the Declaration of Helsinki [35]. Before being enrolled for the study, all the participants’ legal guardians signed an informed consent form.

To be enrolled in the study, participants were required to be aged between 10 and 18 years and present with paramorphism or dysmorphism (i.e., IS). The exclusion criteria comprised patients with secondary scoliosis (e.g., neurological forms) and those with a history of previous surgical interventions involving the spinal column.

### 2.2. Clinical Assessments

**Figure 1:**
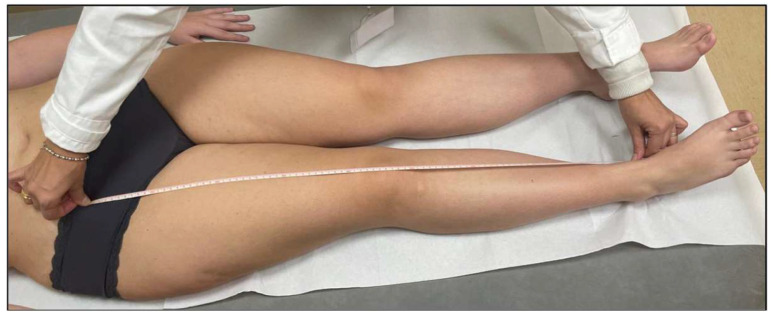
LLD clinical assessment—direct method.

Although radiographic imaging is the most reliable method for measuring lower limb length, clinical measurements were preferred to avoid exposure to ionizing radiation [36,37]. LLDs were clinically assessed using the direct method, measuring the distance between the anterior superior iliac spine and the medial malleolus with a tape measure (Figure 1).

**Figure 2:**
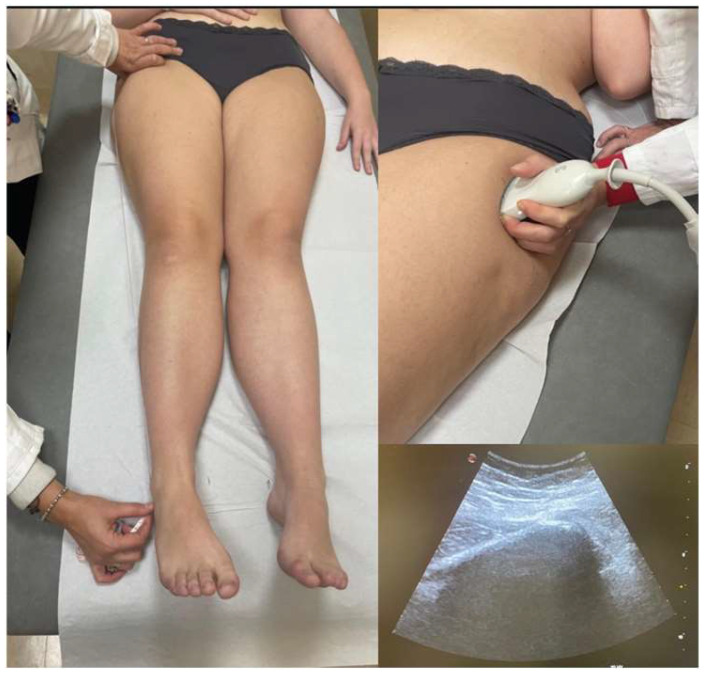
LLD clinical assessment—direct method between greater trochanter and the lateral malleolus, with ultrasound imaging aid.

To enhance the reliability of clinical assessment, an additional direct measurement method was implemented, focusing on the distance between the greater trochanter and the lateral malleolus. In cases where the anatomical identification of the greater trochanter proved challenging, ultrasound imaging was utilized in the clinical setting to improve the landmark localization accuracy (Esaote MyLab 50, Genova, Italy) (Figure 2).

IS and paramorphisms were evaluated through clinical and radiological assessments, including full-spine anteroposterior and lateral radiographs. During the physical examination, patients were assessed in a standing position. In the frontal plane, evaluations focused on asymmetries of the bisacromial line, waist triangles, scapular positioning, and bisiliac line. In the sagittal plane, assessments identified alterations such as lumbar hyperlordosis, thoracic hyperkyphosis, or flat back syndrome. A plumb line was used to measure the sagittal vertical alignment, sagittal spinal curves, and the Sagittal Index. The Adams Forward Bend Test was performed to detect rib humps indicative of vertebral rotation. Radiographic analysis enabled the characterization of the curve type (single, double, or broad-based), curve convexity (i.e., right or left), affected spinal segment, apical vertebra, curve severity (Cobb angle quantification), compensatory secondary curves, and skeletal maturity (assessed via the Risser scale).

BA was also clinically assessed, and data about the presence of asymmetry were recorded. Objective clinical methods for measuring breast volume, such as those based on Archimedes’ principle of water displacement, are challenging to implement in routine practice [38]. Consequently, a subjective visual evaluation was employed, performed with the patient in an upright position and hands placed on the hips to minimize the measurement errors arising from altered shoulder posture that could create apparent asymmetry. Two primary techniques for non-technological assisted breast measurement are validated in the literature: the Qiao et al. formula [39], which uses breast radii and mammary projection, and the El-Oteify et al. formula [40], which measures breast circumference. For this study, the El-Oteify method was chosen due to its speed and reliability. This method employs linear measurements of both breasts, including distances from the mid-clavicle to the nipple and inframammary crease, projection, lateral breast crease to nipple, midline to nipple, and breast circumference. To further mitigate subjectivity, despite the standardized technique, two authors (M.R. and I.I.) independently performed measurements. In cases of significant disagreement, a third author (L.C.) intervened.

### 2.3. Statistical Analysis

Descriptive statistics were used to summarize the demographic and clinical characteristics of the study population. Continuous variables, such as age, BMI, and LLD, were reported as means and standard deviations, while categorical variables, including sex, presence of IS or postural scoliotic attitudes, and presence of BA, were presented as absolute frequencies and percentages. All data were initially entered into an Excel spreadsheet (Microsoft, Redmond, WA, USA, Version 16.97.2) for organization and preliminary review.

The prevalence of LLD and BA was calculated for the entire cohort and stratified by a diagnostic group (IS vs. postural scoliotic attitudes). Associations between LLD, BA, and spinal curve characteristics (location, convexity, and severity) were explored descriptively.

## 3. Results

A convenience sample of 44 participants were enrolled for the purpose of this study.

Of the enrolled participants, 26 (60%; 18 females, 70%; 8 males, 30%) were diagnosed with a spinal dysmorphism, specifically adolescent IS, while 18 (40%; 10 females, 56%; 8 males, 44%) presented with a spinal paramorphism (i.e., scoliotic posture).

In our population, 35 (79.5%) participants exhibited LLD; the mean discrepancy was 0.7 ± 0.6 cm, falling within the mild LLD category (<3 cm), at both direct measurements employed.

BA was observed in 8 out of 28 participants (14%) included in the study. Among these cases, four had a larger right breast, and four had a larger left breast, indicating an equal distribution of asymmetry between sides.

Inter-observer reliability for LLD and BA measurements was assessed using the intraclass correlation coefficient (ICC). For LLD measurements, the ICC was 0.94 (95% CI: 0.89–0.97), and for BA assessments, the ICC was 0.91 (95% CI: 0.85–0.95), indicating excellent agreement.

Demographic, spinal pathology, and asymmetry data of the sample (i.e., sex, age, and BMI) are reported in Table 1.

### 3.1. Scoliotic Posture

Among the 18 participants with postural scoliotic attitudes, the distribution of spinal involvement was as follows:

- Dorsal spine: Ten (56%) participants, with right convex curves in seven patients and left convex curves in three;
- Lumbar spine: Seven (39%) participants, characterized by left convex curves in five cases and right convex curves in two;
- Thoracolumbar spine: One (5%) participant, presenting a left convex curvature.

This distribution highlights the predominance of dorsal involvement in postural scoliotic deviations, with right convex curves being more frequent in this region.

In this group, 13 (72.2%) participants exhibited LLD. The right lower limb was longer than the left in 10 patients (77%). Among the seven patients with lumbar postural scoliotic attitudes, five (71.4%) presented with lower LLD. In all cases, the shorter limb was ipsilateral to the convexity of the scoliotic curve, as confirmed by both direct measurement methods.

Lastly, three (17%) participants showed BA.

### 3.2. Idiopathic Scoliosis

Among the 26 cases of IS, the distribution of spinal involvement and curve characteristics was as follows:

- Lumbar spine: Eleven cases (42%), comprising seven single curves and four double curves;
- Dorsal spine: Eight cases (31%), with seven single curves and one double curve;
- Thoracolumbar spine: Seven cases (27%), including four single curves and three double curves.

Regarding curve convexity:

- Dorsal curves were exclusively right convex;
- Lumbar curves were left convex in eight patients (73%);
- Thoracolumbar curves demonstrated left convexity in 57% of cases and right convexity in 43%.

This analysis reveals distinct regional patterns in curve laterality, with dorsal curves favoring right convexity and lumbar curves predominantly favoring left convexity.

The severity of scoliotic curves, quantified by Cobb angle measurements, showed comparable values between right convex and left convex curves. Right convex curves had a mean Cobb angle of 17° ± 5°, while left convex curves averaged 18° ± 4°. Both fall within the mild scoliosis classification (Cobb angles 10–25°).

In this group, 22 (87%) participants exhibited LLD, confirmed by both direct measurement methods. The distribution of LLD was symmetrical (right limb longer: 11 cases, left limb longer: 11 cases). LLD was associated with nine cases (40%) with lumbar curves.

Concurrent BA in patients with IS was found in five (19%) cases, predominantly involving the dorsal spine with right convex curves (four out of five cases), exhibiting Cobb angles between 10° and 20°. The observed BA was in the right breast in three cases and in the left breast in two cases.

## 4. Discussion

This paper aimed at investigating the prevalence and characteristics of LLD and BA in a population of adolescents presenting with spinal abnormalities (i.e., postural scoliotic attitudes or IS), evaluating potential associations between LLD, BA, and spinal abnormalities. Our findings highlight a notably high prevalence of LLD, with 79.5% of participants exhibiting LLD, predominantly in the mild range (mean discrepancy 0.7 ± 0.6 cm). BA was identified in 14% of the overall sample, with a slightly higher occurrence in those with IS (19%) compared to those with postural scoliotic attitudes (17%). The findings also reveal distinct patterns in curve location and laterality, with right convex dorsal curves and left convex lumbar curves predominating. 

LLD features in our cohort are in line with the other literature reports. Knutson’s analysis of radiographically measured anatomic LLD in the general population found a prevalence of approximately 90%, with a mean discrepancy of 0.5 cm [41]. Importantly, Knutson observed that the majority of these discrepancies are clinically insignificant unless they exceed 2 cm, as passive compensatory mechanisms of the pelvis and spine generally accommodate smaller differences without leading to symptoms or functional impairment. Our cohort, with a high prevalence of LLD (79.5%) and a mean magnitude of 0.7 cm, aligns closely with Knutson et al., reinforcing the notion that mild LLD is common in both healthy and clinical populations. Furthermore, Knutson noted a slight predominance of right-sided shorter legs, a pattern also observed in our sample, though the clinical impact of this laterality remains limited in cases of minor discrepancy. These parallels suggest that the high frequency and mild degree of LLD observed in our study reflect the broader epidemiological patterns described in the literature. Recent studies by Kobayashi et al. [42] and Marsiolo et al. [3] provide critical insights that align with and expand upon our results. Kobayashi et al. conducted a retrospective study on pediatric patients with significant LLD (≥2 cm, mean 4.43 cm), demonstrating that a substantial proportion (65%) developed scoliosis, predominantly lumbar and always with convexity toward the short leg side. Importantly, the authors found a strong positive correlation between the severity of LLD and both the Cobb angle and pelvic obliquity, as well as a significant association with vertebral rotation. Kobayashi et al.’s results indicate that while mild LLD is common and often clinically silent, more severe discrepancies (≥3 cm) are associated with the development of structural changes in the spine, including progressive scoliosis and vertebral rotation. Marsiolo et al. focused on a pediatric population with functional scoliosis (Cobb angle <15°), specifically investigating the relationship between LLD, sacral shelf inclination, and vertebral rotation. Their study revealed that even mild LLD (mean 0.6 cm) can be associated with vertebral rotation, with a threshold of 0.5 cm identified as increasing the risk of both sacral shelf inclination and vertebral rotation. They demonstrated a linear relationship: for each millimeter of LLD, there was a corresponding increase in sacral shelf inclination (0.03 cm) and a measurable increment in vertebral rotation (0.12°). Contrary to the traditional view that only large discrepancies are clinically relevant, these findings suggest that even small LLDs may induce subtle but potentially significant changes in spinal alignment and mechanics, especially during the growth period. Marsiolo et al. also propose that the early identification and management of LLD, even at low thresholds, could be important to prevent the development or progression of structural changes, including vertebral rotation, in growing children. Thus, it appears that the prevalence of LLD in adolescents with spinal asymmetries is in line with the high rates reported in the general pediatric population, but that the clinical impact may be modulated by the degree of discrepancy. While Knutson’s review [41] emphasizes the benign nature of mild anatomical LLD in most individuals, the evidence from Kobayashi and Marsiolo underscores that even small discrepancies may have biomechanical consequences in susceptible populations, particularly during skeletal growth. Our findings of a high prevalence of mild LLD (mean 0.7 cm) and its association with postural and structural spinal changes are thus corroborated by these studies, supporting a nuanced view about LLD and spinal morphology and function in the pediatric age group. These findings reinforce the importance of comprehensive clinical and, when indicated, radiological assessment of LLD in children and adolescents presenting with spinal asymmetries. They also suggest that individualized management strategies, including early intervention for even mild discrepancies, may be warranted to mitigate the risk of progression to structural scoliosis and vertebral rotation, particularly in those with additional risk factors or signs of compensatory spinal changes.

In the subgroup of patients with a lumbar scoliotic posture, our findings revealed a possible biomechanical association between LLD and the postural deviation of the spine. Specifically, in five out of the seven observed cases, the shorter lower limb was located on the side of the convexity of the lumbar curve. This observation supports the hypothesis that the scoliotic posture in these patients may represent a compensatory response to pelvic tilt in the frontal plane, directly induced by LLD. While our study’s design does not allow for conclusive assumption, this pattern is consistent with the biomechanical model described by Gurney, who emphasized that pelvic obliquity resulting from LLD can lead to a compensatory lumbar curve, with the convexity typically oriented toward the short leg side [24]. In contrast, among subjects with adolescent IS, no direct correlation was identified between LLD and the location or laterality of the curve. The recent study by Mishra et al. reinforces this concept by demonstrating that while LLD is relatively common in IS populations, its magnitude is typically mild and not systematically associated with the side or region of the spinal curve. In their cohort of 141 patients, the majority exhibited either no significant LLD or only minor discrepancies, and there was no evidence that LLD influenced the pattern or progression of the scoliotic curve. Mishra et al. further emphasized that, although functional scoliosis can result from more pronounced LLD in IS, the presence of LLD is more likely to be incidental or to add a minor functional component to an already established structural deformity, rather than serving as a primary etiological factor. They advocate for routine screening of LLD in IS to rule out any functional contribution, but highlight that the correction of mild LLD does not typically alter the course of structural scoliosis [43]. Crijns et al. provide additional mechanistic insight, proposing that the origin of IS lies in restrained differential growth between the vertebral column and surrounding musculo-ligamentary structures, rather than in peripheral asymmetries such as LLD. Their experimental model shows that three-dimensional spinal deformity characteristic of IS can develop in the absence of any initial left–right asymmetry, driven instead by imbalances in growth dynamics and tension within the torso. This supports the view that IS is primarily a disorder of intrinsic spinal and paraspinal growth regulation, with LLD playing, at most, a secondary or coincidental role [44]. Consistent with the literature, no correlation was found between the severity of the curve (expressed by the Cobb angle) and the presence or magnitude of LLD. This implies that more pronounced scoliotic curves are not necessarily associated with greater lower limb length discrepancy, nor does the presence of LLD influence the severity of the curve [45,46].

The relation between BA and spinal abnormalities has a growing presence in the literature. Denoel et al. provided important insights, especially in adolescent females [47]. These authors assessed 24 women with right thoracic IS using clinical examination, anthropometric measurements, and 3D surface scanning. Their findings revealed a consistent pattern: the right breast was smaller and positioned higher in the majority of cases, correlating with the side of the scoliotic convexity. Specifically, 19 out of 24 patients had a smaller right breast, and 20 had a higher right breast, matching the typical right convex thoracic curve seen in IS. These results were robust across both clinical and 3D morphometric assessments, supporting the reliability of meticulous physical examination for detecting BA in this population. Denoel et al. also emphasized that BA in IS is not solely a matter of breast tissue volume, but is closely linked to thoracic cage deformation caused by the underlying spinal curvature. The presence of an anterior rib hump, especially beneath the left inframammary fold, was identified as a key clinical sign of this association. Importantly, the study demonstrated that BA in IS is often lateralized (commonly right-sided in right thoracic curves), and that BA should prompt clinicians to consider underlying scoliosis, especially when associated with other signs such as rib prominence or mammary dystopia. These findings are consistent with the recent literature. For example, Ramsay et al. found that BA is significantly more common in IS than in the general population, and that the degree of asymmetry may correlate with curve severity, though they did not report a constant linear relationship [25]. Similarly, Chan et al. used CT morphometric analysis and patient-reported outcomes to show that BA is prevalent in IS patients with structural thoracic curves, with the breast on the convex side typically smaller and positioned higher [31]. Notably, while some studies report a correlation between BA and curve severity (Cobb angle), others suggest that BA may persist independently of curve magnitude, especially in milder cases [30]. The study by Duygu et al. provides further evidence supporting the association between BA and IS, particularly in adolescent females with thoracic curves. Their research demonstrated that the breast on the convex side of the thoracic scoliosis was consistently smaller in volume, had a shorter mammary base, and a higher nipple position compared to the contralateral side. These findings were attributed to the deformation of the rib cage and the resulting changes in the anterior chest wall mechanics caused by the underlying spinal curvature. Duygu et al. emphasized that such structural alterations, rather than intrinsic differences in breast tissue, are the primary drivers of BA in this context [48]. However, our findings diverge from this typical pattern. In our population, the direction and degree of BA did not consistently correspond to the convexity of the scoliotic curve. This discrepancy suggests that compensatory mechanisms—such as variations in soft tissue adaptation, postural adjustments, or even differences in growth patterns—may modulate the expression of BA in individual patients [49,50,51]. Additionally, the subjectivity inherent in clinical measurement and assessment techniques may contribute to variability in reported asymmetry, as highlighted by Duygu et al., who noted the importance of standardized and objective methods for evaluating BA in scoliosis.

Considering the impact of LLD and BA in people with IS, appropriate treatment strategies seem crucial. In rehabilitation, Individual Rehabilitation Projects (IRPs) are structured, patient-specific plans developed by multidisciplinary teams to address disabilities of various kinds, comprising the complex picture of IS [52,53,54]. IRPs focusing on trunk symmetry and shoulder alignment are critical for patients with IS. IRPs should comprise programs incorporating scoliosis-specific therapeutic exercises, which have been established as beneficial in addressing deformities through tailored interventions. A systematic review highlighted the effectiveness of such therapies in enhancing posture and reducing curvature among adolescents with IS, emphasizing the challenges posed by breast asymmetry during treatment [55]. Additionally, continuous monitoring and modification of rehabilitation strategies according to individual progress are essential to prevent worsening asymmetries that could impact the patient’s self-perception and overall Quality of Life (QoL) [56]. Developing an IRP for individuals with IS and LLD is essential for optimizing spinal alignment and overall function. The rehabilitation protocol must take into account specific factors related to scoliosis, such as curvature severity and skeletal maturity, as well as the nature and extent of any LLD. A thorough assessment should be conducted at the outset. This evaluation includes measuring spinal curvature using Cobb angle assessments, assessing leg lengths using both clinical and imaging methods, and evaluating pelvic alignment [57]. Specific therapeutic exercises should be tailored to address LLD; specific exercises can help promote pelvic leveling, which may alleviate compensatory curvature in the spine. Exercises focused on strengthening the core, back muscles, and hip stabilizers can enhance strength and improve overall posture [58]. Foot orthotics, such as shoe lifts, should be considered for patients with significant LLD (typically exceeding 1 cm) to correct pelvic tilt and reduce functional scoliosis [59]. Incorporating regular physical activity is vital. Proactive involvement in sports and physical exercises has been shown to decrease the risk of curvature progression in adolescents with IS [60]. Accordingly, BA in patients with IS is a clinically significant issue that can have a profound impact on the patient’s QoL, self-image, and psychological health [58,61]. This morphological alteration often manifests as cosmetic concerns, where the asymmetric appearance can lead to decreased self-esteem and body image dissatisfaction [62]. Therapeutic exercise and bracing have shown promise in reducing the curvature of the spine and the associated BA [55,63]. Furthermore, the QoL in patients with scoliosis has been reported to correlate negatively with the degree of spinal curvature and resultant physical asymmetries, suggesting that early and comprehensive management can improve both functional outcomes and emotional health [64]. Thus, specific exercises designed to address trunk balance and symmetry during critical growth periods are essential to mitigate the potential for progressive asymmetry [54].

### Limitations

Our study does not come without limitations. We enrolled a relatively small sample size, which limits the generalizability of the findings. The population was restricted to a single outpatient unit in Rome, Italy, which may introduce geographic or referral bias and limit external validity.

Although the study used direct clinical methods and, when needed, ultrasound to aid anatomical localization, the clinical measurement of LLD may be prone to subjective errors. Moreover, subjective assessment of BA, even if carried out through standardized techniques, can miss subtle asymmetries. However, the decision to use clinical (rather than radiographic) methods for LLD measurement is justified by the desire to avoid unnecessary radiation exposure in a pediatric population. We tried to enhance reliability by using two direct measurement techniques and supplementing them with ultrasound when anatomical landmarks were difficult to identify. For subjective measures like BA, we employed a validated technique and two independent assessors, with a third involved in cases of disagreement. However, these subjective measurements reduce the reliability of our results, which should be interpreted with caution. Future research should focus on objective radiographic measurements using innovative technologies (e.g., EOS imaging or MRI 3D scans).

Lastly, the study has a cross-sectional and observational design, which precludes any conclusions about causality or the temporal relationship between LLD, spinal abnormalities, and BA. Longitudinal data would be necessary to determine whether LLD precedes or results from spinal changes, or whether BA develops as a consequence of scoliosis progression.

## 5. Conclusions

This study demonstrates a high prevalence of mild LLD and BA among adolescents with spinal paramorphisms and IS. LLD was present in nearly 80% of participants, predominantly within the mild range, and was often associated with postural abnormalities, particularly in cases of lumbar postural curves where the shorter limb corresponded to the curve convexity. However, in IS, LLD did not show a consistent relationship with curve location, laterality, or severity.

BA was observed in a minority of cases, with a slightly higher prevalence in IS compared to postural scoliotic attitudes. The direction and magnitude of BA did not consistently align with the convexity of the spinal curve, indicating that BA in this population may be influenced by a combination of thoracic cage deformation, individual growth patterns, and compensatory mechanisms.

Overall, the findings suggest that while mild LLD and BA are frequent in adolescents with spinal asymmetries, their clinical significance is generally limited, especially in the absence of more severe discrepancies. Comprehensive clinical assessment remains essential for early identification and individualized management. Future research with larger, longitudinal cohorts and objective measurement techniques is warranted to clarify the causal relationships and long-term implications of these asymmetries in pediatric populations with spinal deformities.

## Figures and Tables

**Table 1:** Demographic, spinal pathology, and asymmetry data.

Category	Details
**Total Participants**	44
**Sex Distribution**	28 females (63.6%), 16 males (36.4%)
**Mean Age**	13.7 ± 2.3 years
**Mean BMI**	20.6 ± 3
**Spinal Deformities**	
**Dysmorphism (AIS)**	26 (60%): 18 females (70%), 8 males (30%)
**Paramorphism**	18 (40%): 10 females (56%), 8 males (44%)
**Lower Limb Discrepancy**	
**Prevalence**	35 (79.5%)
**Mean Discrepancy**	0.7 ± 0.6 cm (mild LLD: <3 cm)
**Breast Asymmetry**	8/28 females (14%): 4 right larger, 4 left larger

## Data Availability

Data are available upon reasonable request to the corresponding author.
